# Supplementary material for: Type-H endothelial cell protein Clec14a orchestrates osteoblast activity during trabecular bone formation and patterning
Source: Commun Biol. 2024 Oct 11;7:1296. doi: 10.1038/s42003-024-06971-3 (PMC11470016; doi:10.1038/s42003-024-06971-3)
Supplement: Supplementary file 2 — Supplementary Information [file 42003_2024_6971_MOESM2_ESM.pdf]

Supplementary Figure 1. Increased expression of *Clec14a* and *Mmrn2* in bone type-H endothelial cells.

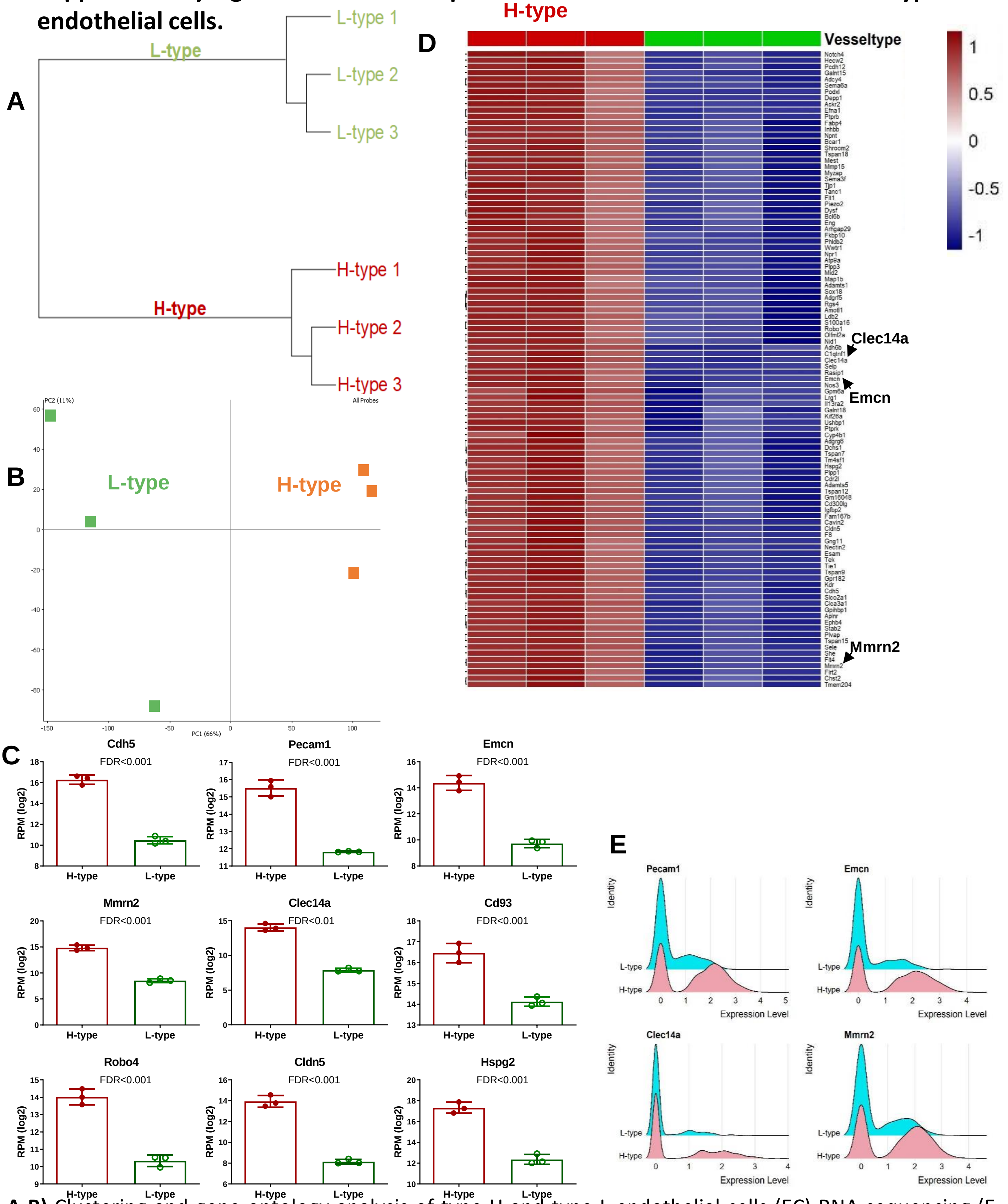

A-B) Clustering and gene ontology analysis of type-H and type-L endothelial cells (EC) RNA sequencing (E-MTAB-4066 dataset). Unsupervised hierarchical clustering of log2 normalized counting reads over exons (RPM) for all detected genes. **C)** Scatter plots of log2 normalized counts for key gene EC transcripts and Clec14a interacting partners, identified as differentially expressed between H- (red) and L-type (green) EC. **D)** Heatmap illustrations of Z-score normalized gene expression values for differentially expressed genes in tibia derived H- and L-type EC isolated from postnatal day 6 pups. Heat map displays data for genes upregulated (red) in H-type EC. Black arrows indicate genes of interest – *Clec14a*, *Mmrn2* and *Emcn*. **E)** Sc-RNA sequencing (GSE128423 dataset) plots showing the increased mRNA expression of type-H EC markers *Pecam1 and Emcn,* as well as *Clec14a* and *Mmrn2* in type-H versus type-L EC.

Supplementary Figure 2. Characterisation of type-H vessel density in neonatal and juvenile wildtype and *Clec14a*$^{-/-}$ mice.

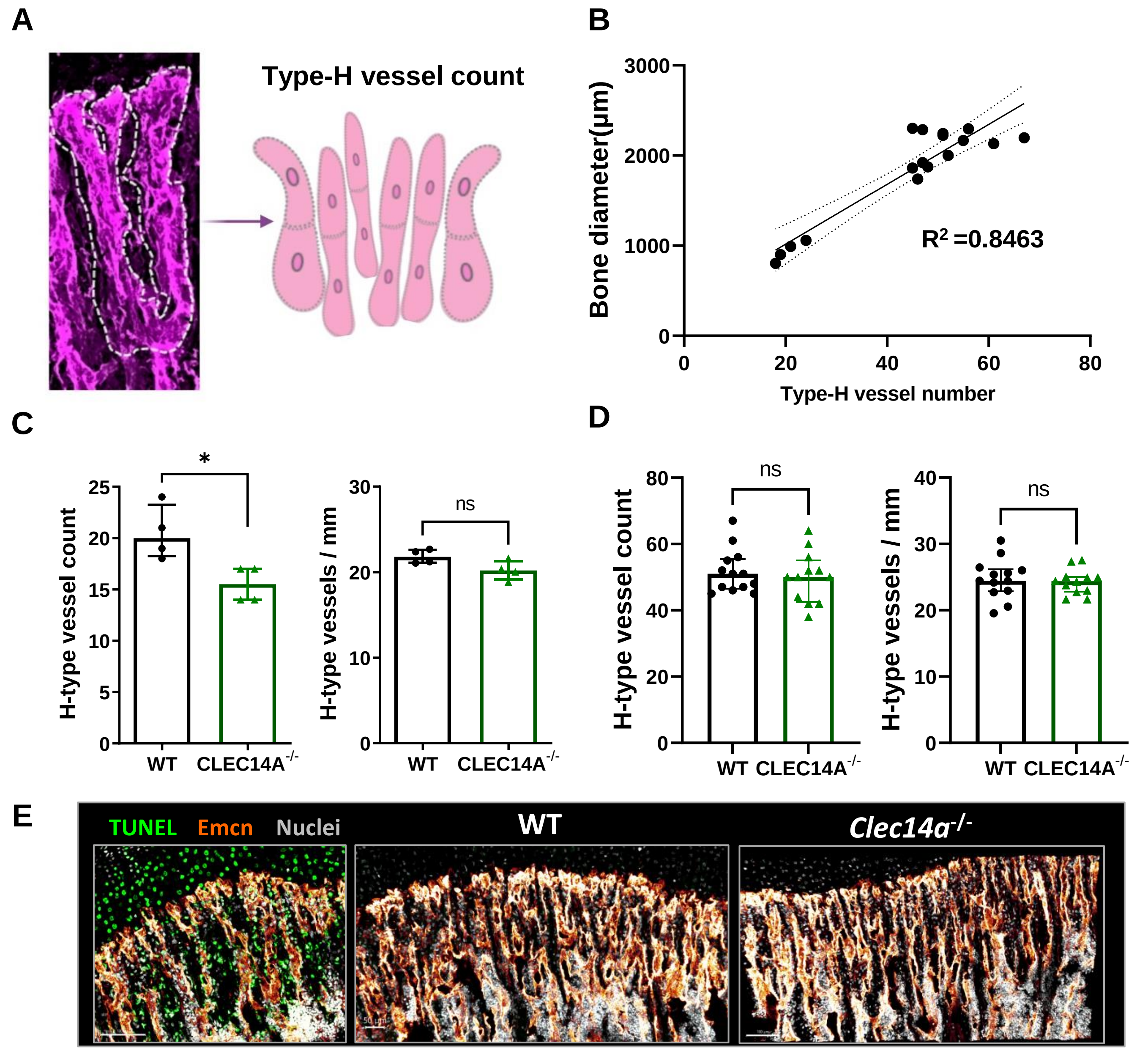

A) Schematic representation illustrating the analysis technique utilised to count type-H vessels at the actively expanding vascular front in the proximal end of the tibia metaphysis. Dashed line contours two type-H vessels which are untied at their distal ends at a branching point. **B)** Scatter plot showing the association between type-H vessel number and bone diameter. Data was analysed with a simple linear regression test, $p < 0.0001$**.** **C-D)** Quantification of H-type columns (left graph of pair) and their calculated density in at the metaphysis leading edge (right graph of pair) at **C)** postnatal day 4 and **D)** 4-weeks of age. Data is represented as median ± IQR, results were analysed with a Mann-Whitney test. **E)** Immunofluorescent imaging of apoptosis in the 4-week-old mouse tibial metaphysis (TUNEL - green). Vasculature immunolabelled using Emcn (orange), nuclei Hoechst (grey). Left panel shows a positive control produced by treatment of slide with DNAse I. Scale bars are 100 µm for positive control and 50 µm for experimental images.

Supplementary Figure 3. High-resolution 3D confocal microscopy of the neonatal *Clec14a*$^{+/+}$ and *Clec14a*$^{-/-}$ mouse tibia vascular network.

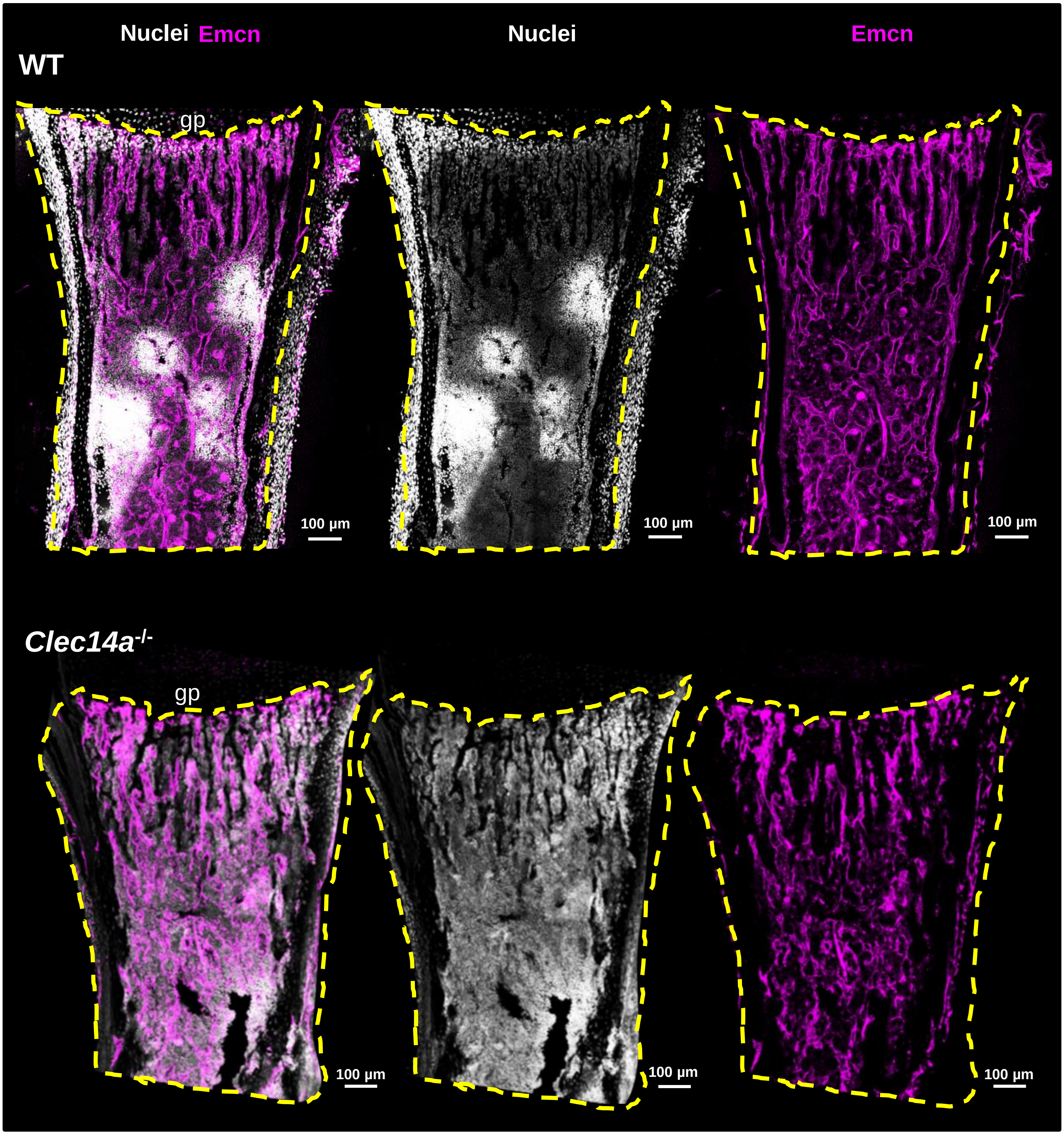

Representative maximum intensity projection tile scan images show no striking differences between the *Clec14a*$^{+/+}$ and *Clec14a*$^{-/-}$ vascular networks of postnatal day 4 (P4) mouse pups. Vasculature immunolabelled with anti-Emcn antibody (magenta), nuclei were stained with Hoechst (grey). Yellow dashed line demarcates the vascular area encased in the cortical bone envelope interior to the bone endosteum and demarcates the growth plate (avascular) – top metaphysis boundary. Left image = merged. Middle image = nuclei only. Right image = Emcn only. Scale bars are 100 µm. Abbreviations: gp-growth plate

Supplementary Figure 4. Characterisation of type-H vessels in neonatal and juvenile *Clec14a*$^{-/-}$ and *Clec14a*$^{+/+}$ mice.

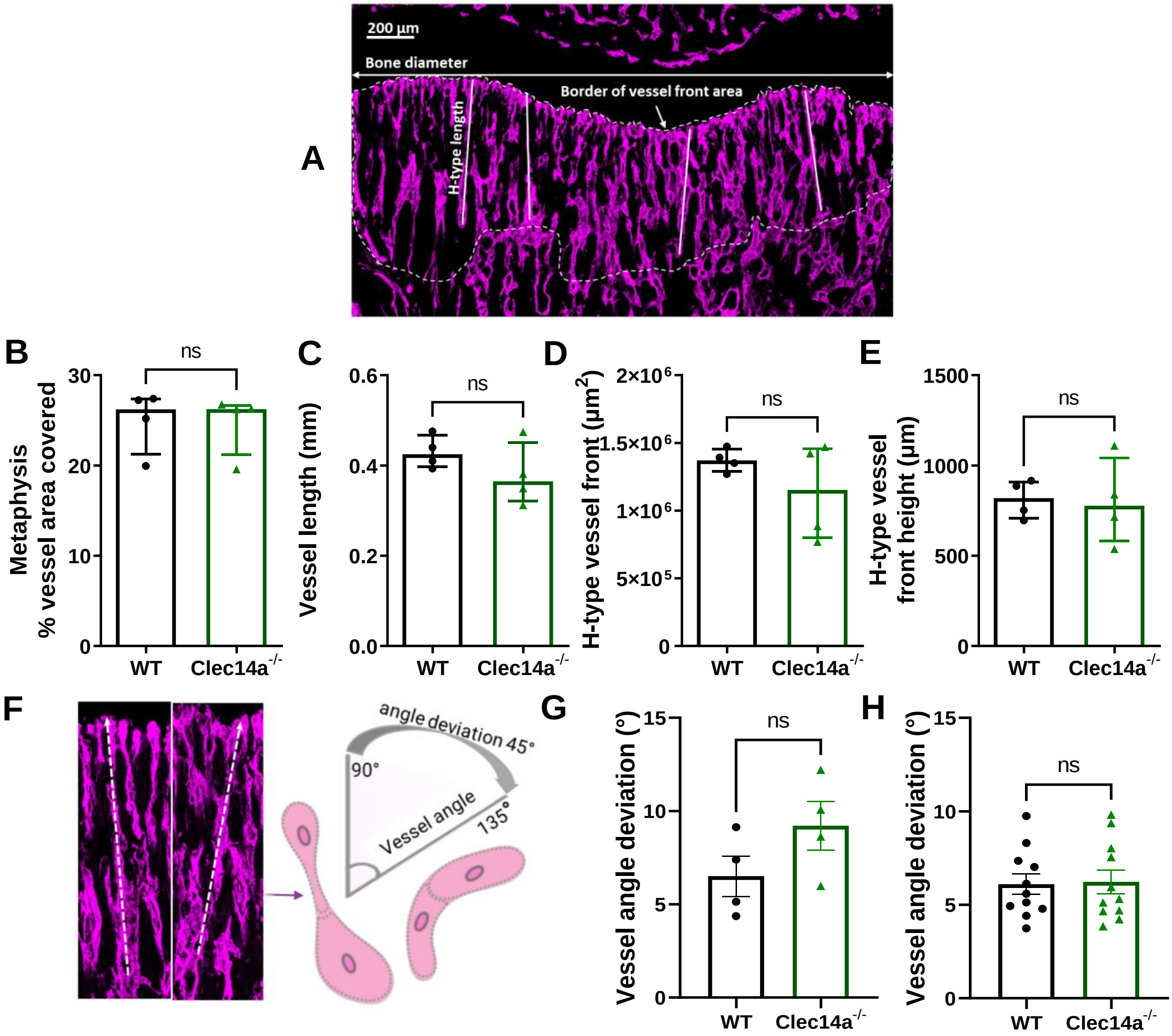

A) Schematic diagram illustrating the analysis strategy employed to characterize the H-type vascular front in P4 male mouse tibiae, the area analysed encompassed all vessels from the superior end of the metaphysis to the transition zone, this area was delineated by drawing around the cranio-latero caudal borders of parallel H type columns. **B)** Quantification of the % area covered by Emcn immunolabeled vessels in the tibia metaphysis. **C)** Quantification of the length of H type columns in the metaphysis, data represents mean values for each biological replicate obtained from 7-11 vessel measurements in each image **D)** Measurement of the area covered by the H-type vessel front. **E)** Quantitative evaluation of the length of the H type vessel front. Data is represented as median ± IQR, results were analysed with a Mann Whitney test. No significant differences were detected (ns)**.** **F)** Schematic representation illustrating the measurement technique utilised to assess the directional distribution of type-H vessels at the actively expanding vascular front at the proximal end of the tibia metaphysis. Vessel orientation was measured using the straight-line tool in Image J, vessel angle deviation was calculated as the absolute value deviation from 90° (arc degrees). **G-H)** Quantification of type H-vessel angle deviation in **G)** neonatal pups (P4) and **H)** juvenile mice (4 weeks-old). Data is represented as median ± IQR, results were analysed with a Mann-Whitney test.

Supplementary Figure 5. Distribution of osteoblasts in neonatal (P4) *Clec14a*$^{+/+}$ and *Clec14a*$^{-/-}$ murine tibia.

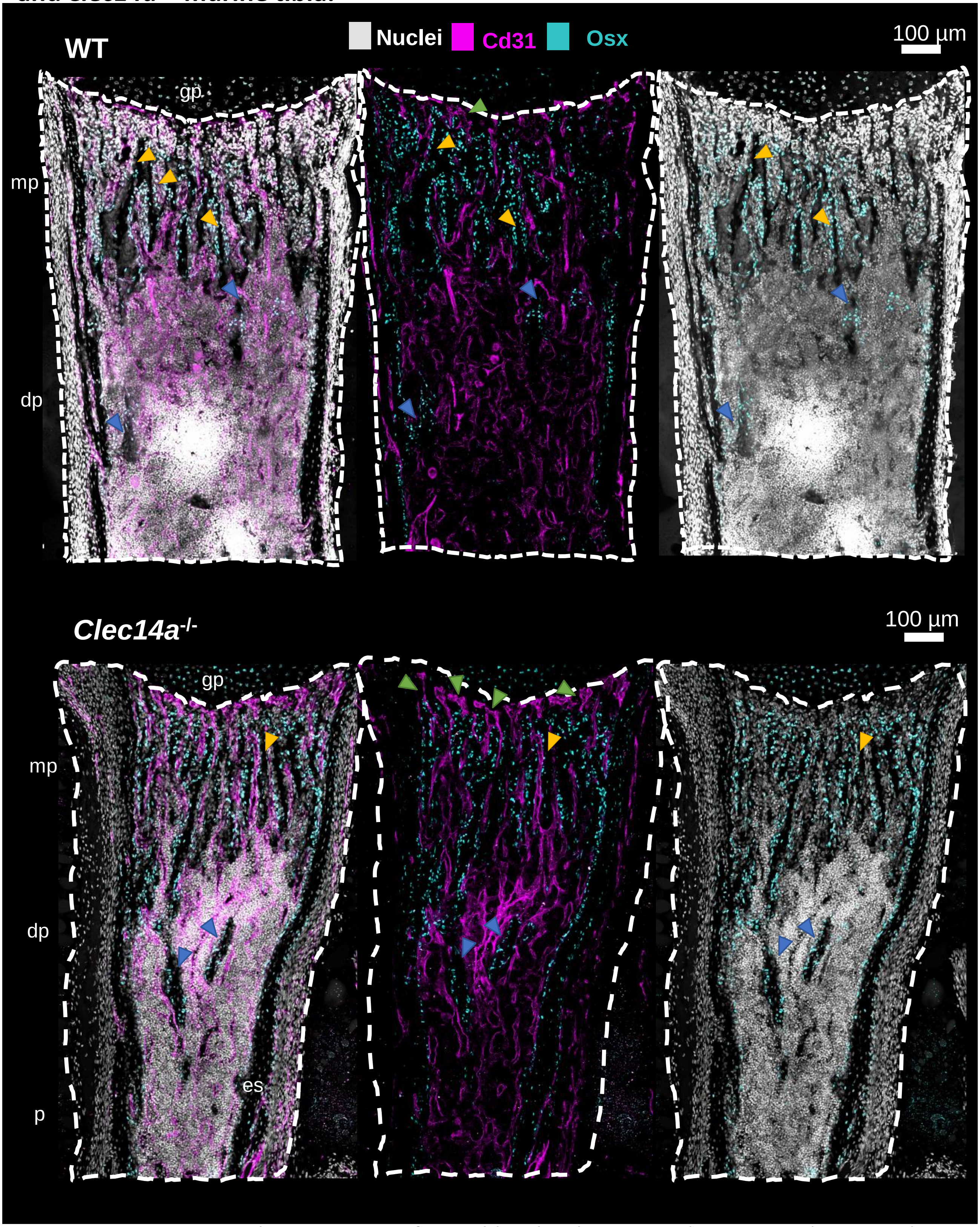

Representative images show mapping of osteoblast localisation in the murine tibia. Vessels are labelled with Cd31 (magenta), whilst osteoblasts are identified by their expression of Osx (cyan), the entire tissue section is counterstained with Hoechst to identify nuclei (grey). Note the limited localisation of Osx^{+} cells around type-H vessels in the metaphysis and endosteum. Blue arrowheads indicate bone pits void of vasculature with osteoblasts distributed around the exterior edge of the bone trabeculae, yellow arrow heads indicate areas where osteoblasts are near columnar type-H vessels, and green arrow heads indicate areas at the leading front of the expanding vasculature which are void of osteoblast coverage. Abbreviations: dp-diaphysis, gp-growth plate, mp-metaphysis, es-endosteum, p-periosteum. Scale bars are 100 µm.

Supplementary Figure 6. Distance of Osx$^+$ cells from vessels in the murine tibia metaphysis and diaphysis in juvenile (4-week-old) Clec14a$^{+/+}$ and Clec14a$^{-/-}$ male

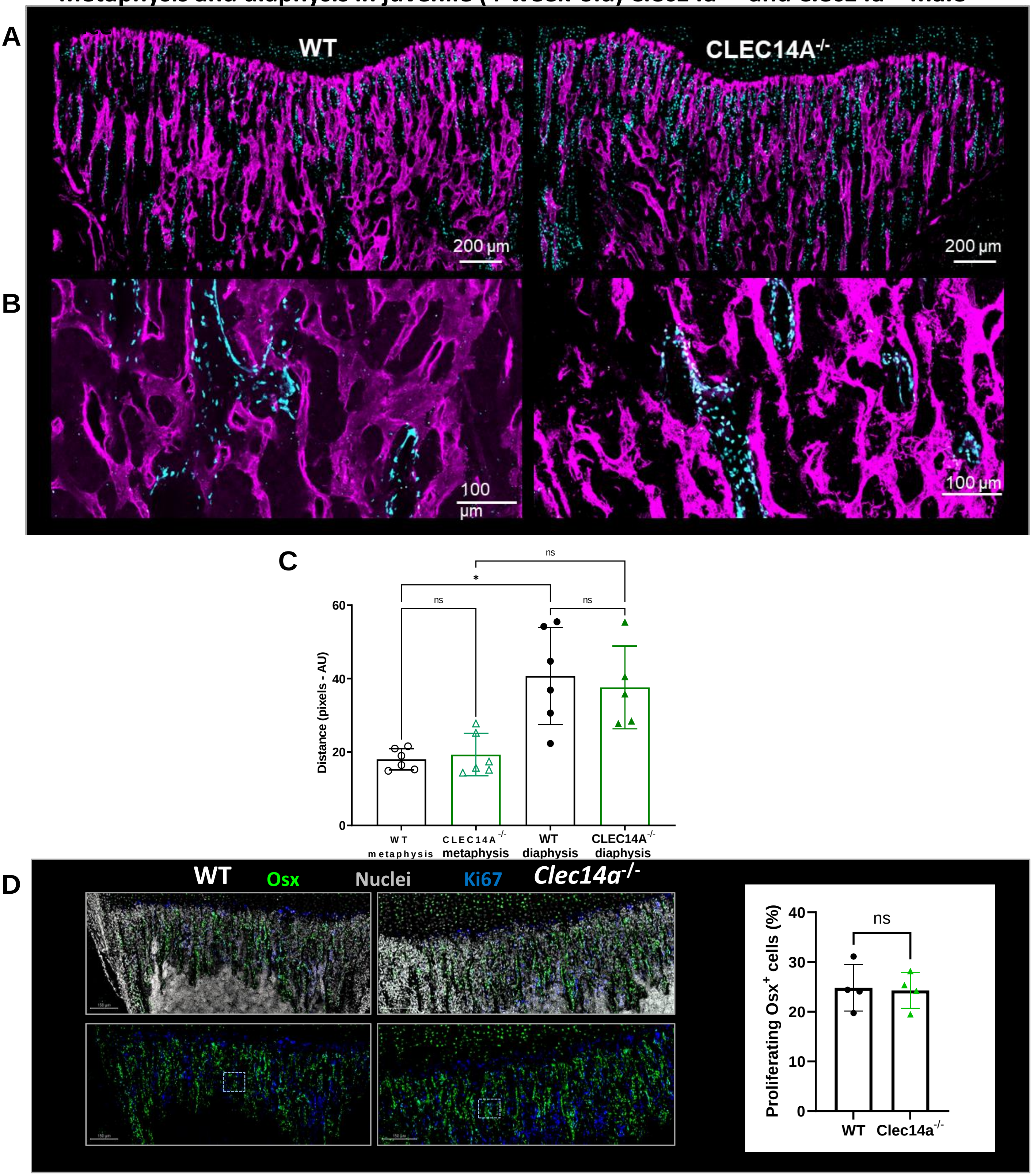

Distance of Osx$^+$ cells from vessels in the murine tibia metaphysis and diaphysis in juvenile (4-week-old) Clec14a$^{+/+}$ and Clec14a$^{-/-}$ male mice. Representative images of **A)** metaphysis and **B)** diaphysis regions. Bone sections were stained with CD31 (magenta) and Osx (cyan). Scale bars are 200 µm (A) and 100 µm (B). **C**) Quantitation of distance of Osx$^+$ cells from centre of the nearest neighbouring vessel shows a significant distancing from vessels in the diaphysis region compared to the metaphysis. Data is represented as Mean ± SD, N = 6 for all groups, results were analysed with a Kruskal-Wallis test, * = $P < 0.05$, ns = not significant. **D)** Representative images showing no difference in proliferation between Clec14a$^{+/+}$ (WT) and Clec14a$^{-/-}$ bone cells in the 4-week old mouse metaphysis. Results were analysed with a Mann-Whitney test.

Supplementary Figure 7. Cortical parameters in *Clec14a*$^{-/-}$ vs *Clec14a*$^{+/+}$ male mice tibial midshaft.

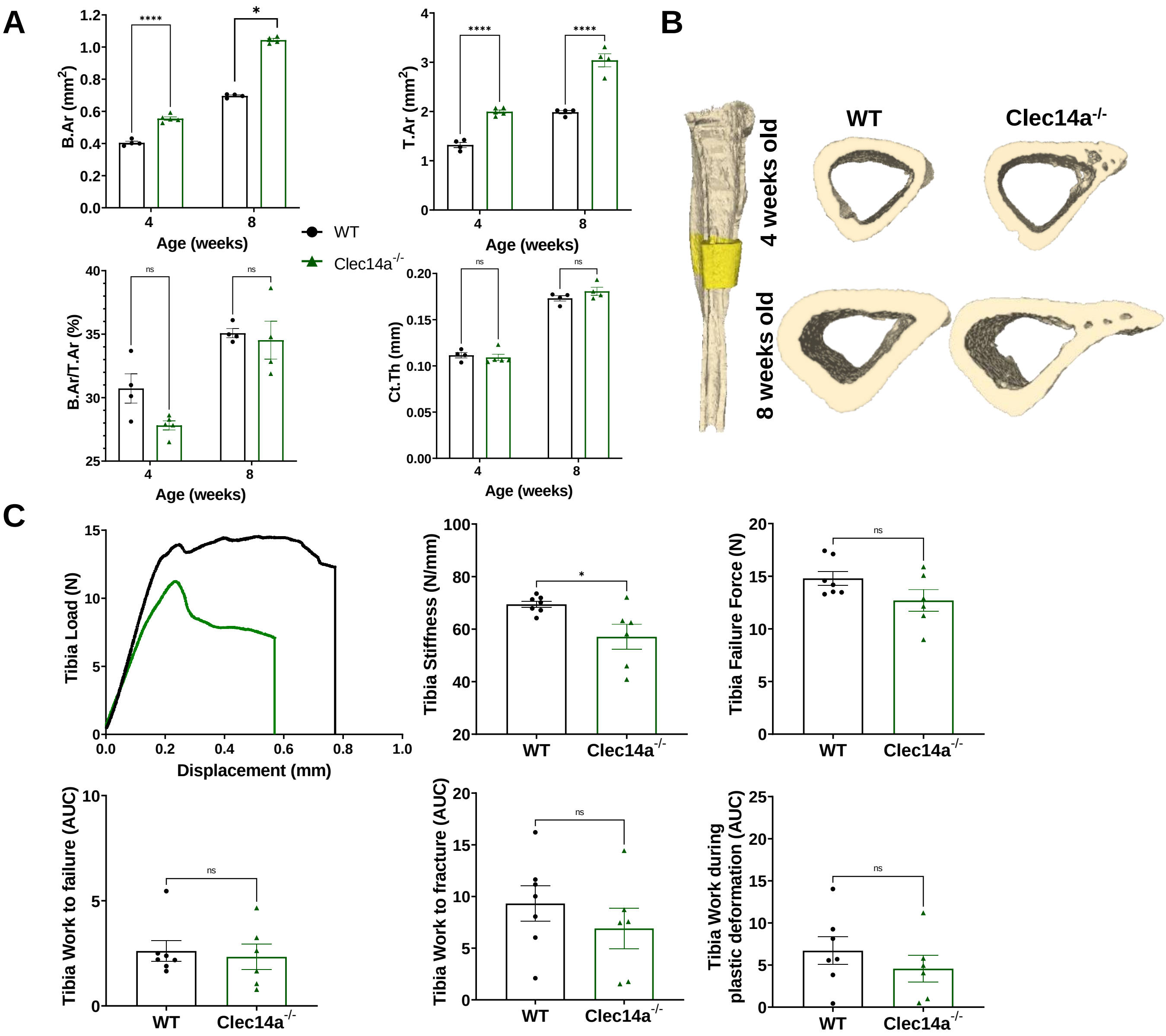

A) Micro-computed tomography (μCT) analysis of bone cortical parameters in the male tibia (Cortical Bone Area-B.Ar, Cortical Tissue Area-T.Ar, Cortical Percent Bone Area-B.Ar/T.Ar, and Cortical Thickness-Ct.Th)**,** sample size (n=4). Results are presented as mean ± SEM, data was analysed with one-way ANOVA, followed by Šídák's multiple post hoc, * = $p < 0.05$, ****=$p < 0.0001$. **B)** Representative μCT 3D volume renderings of cortical bone from 4- and 8- weeks old *Clec14a*$^{+/+}$ and *Clec14a*$^{-/-}$ male mouse tibias. Images show cortical bone (ivory). **C)** Tibia analysis, top row, left to right: three-point bending load vs displacement graphs of 8-week-old murine tibiae tested to fracture (one representative trace for each genotype); stiffness; force to failure. Bottom row, left to right: work to failure; work to fracture and work during plastic deformation (all three presented as Area Under the Curve: AUC. Results are presented as mean ± SEM, data was analysed with an unpaired t test, * = $p < 0.05$.

Supplementary Figure 8. Trabecular and cortical morphometric changes in the female *Clec14a*$^{-/-}$ tibia.

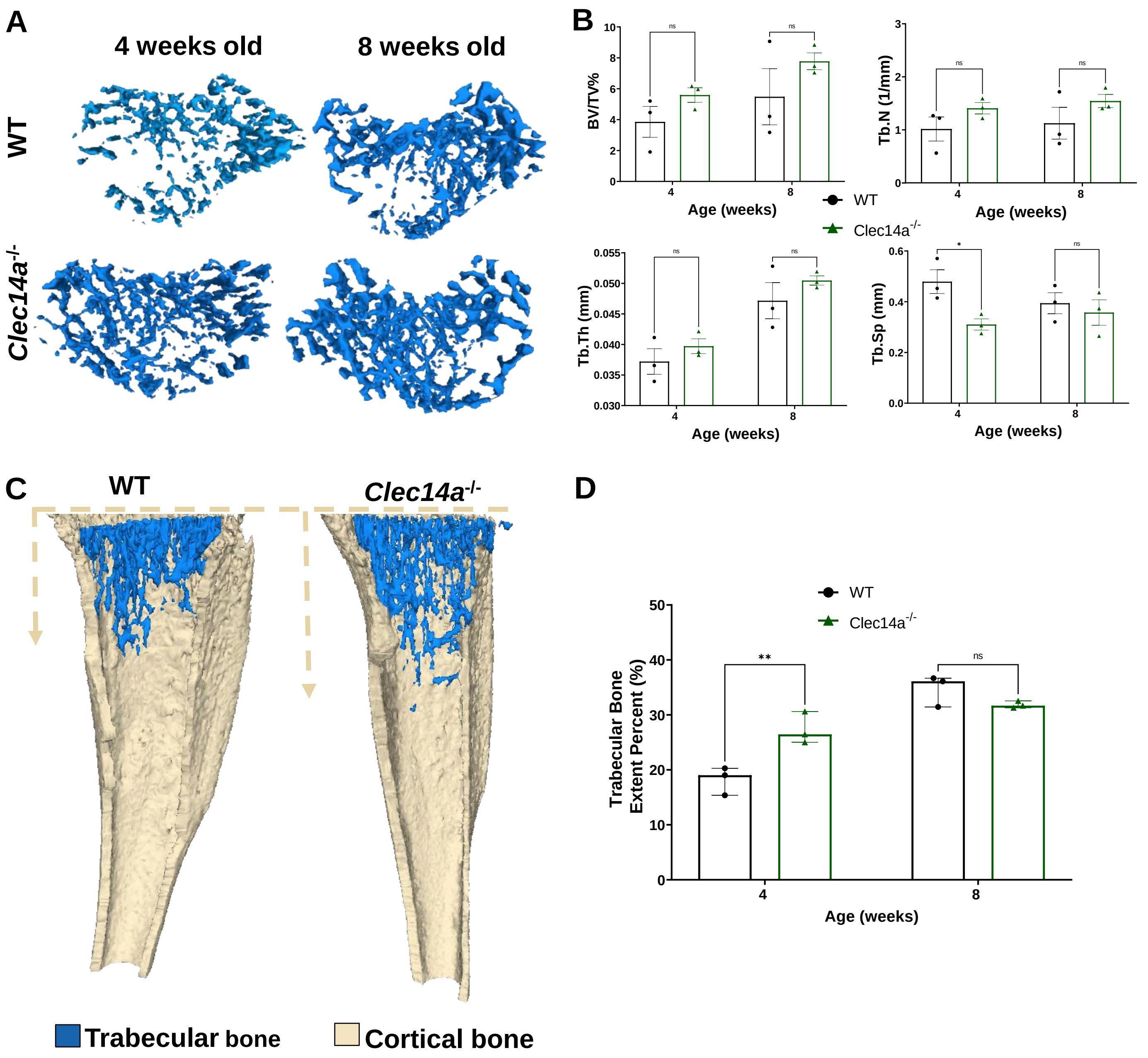

A) Representative µCT analysis sagittal images starting at the end of the proximal growth plate of 4- and 8- weeks old *Clec14a*$^{+/+}$ and *Clec14a*$^{-/-}$ female mouse tibias. Images show trabecular bone (blue) density. **B)** Micro-computed tomography (µCT) analysis of bone trabecular parameters in the proximal tibial metaphysis. **C)** Representative µCT sagittal plane images of trabecular bone extent of 4-weeks old *Clec14a*$^{+/+}$ and *Clec14a*$^{-/-}$ mouse tibiae. Images show trabecular bone extent (blue) and the surrounding cortical bone (ivory). **D)** Extension of trabecular bone (Trabecular Bone Extent Percent) measured from the inferior part of the proximal growth plate to the most distal point in space occupied by trabecular bone towards the diaphysis *Clec14a*$^{+/+}$ vs *Clec14a*$^{-/-}$ female mice. Results are presented as median ± IQR, data was analysed with multiple Mann Whitney tests, ** = $p < 0.01$, sample size (n=3).

Supplementary Figure 9. Expression of genes regulating bone formation and growth.

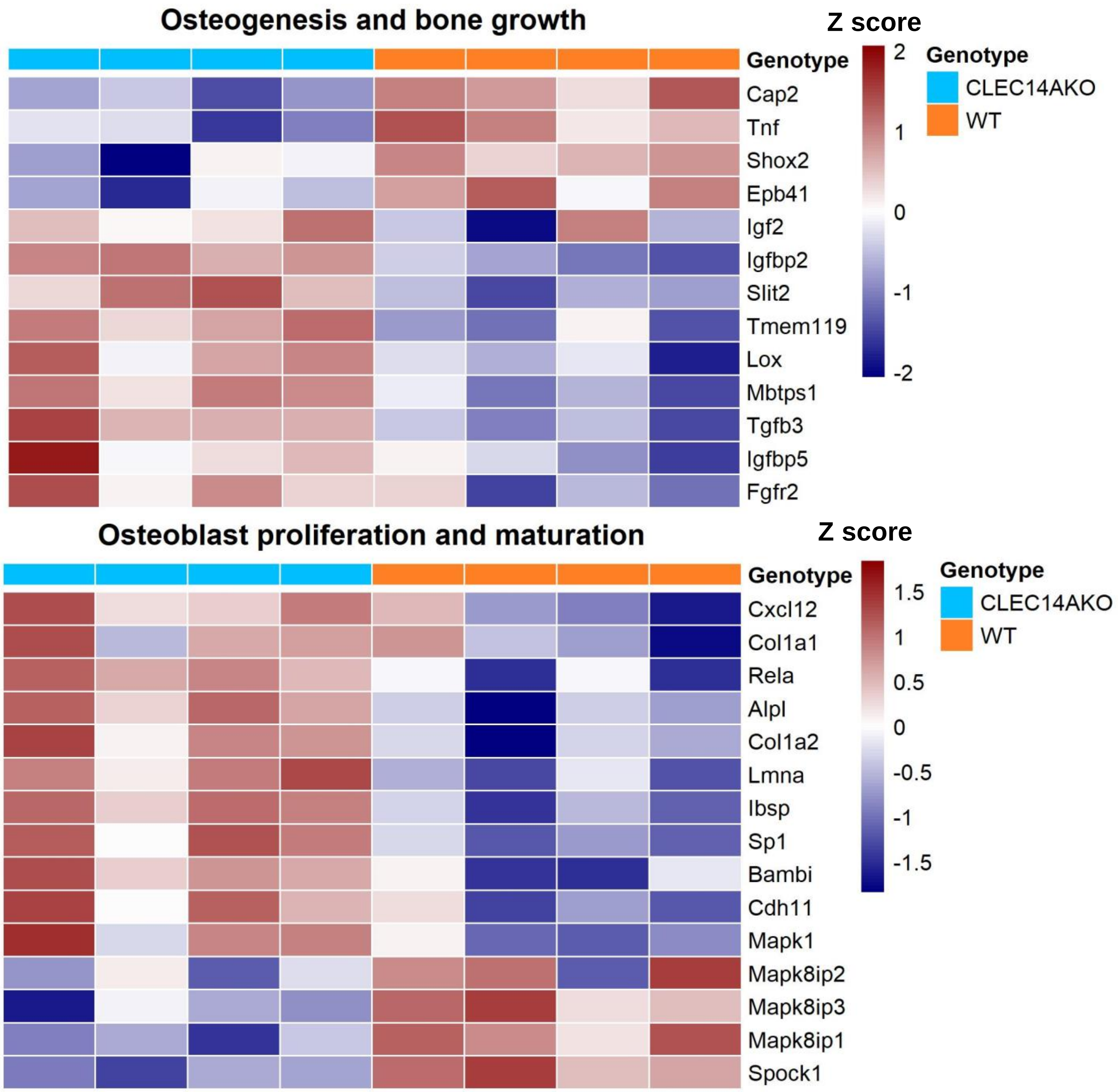

A) Heatmap presents normalised (Z score) gene expression patterns for osteogenesis and bone growth transcripts differentially expressed in *Clec14a*$^{-/-}$ samples. **B)** Heatmap presents normalised (Z score) gene expression patterns for transcripts for molecules moderating osteoblast proliferation and maturation. Transcripts presented in all heatmaps were identified as statistically significant by DESeq2 analysis, padj <0.05.

Supplementary Figure 10. Flow cytometry gating strategy of bone cells isolated from the tibia and femora of P14 *Clec14a*$^{-/-}$ and *Clec14a*$^{+/+}$ mice.

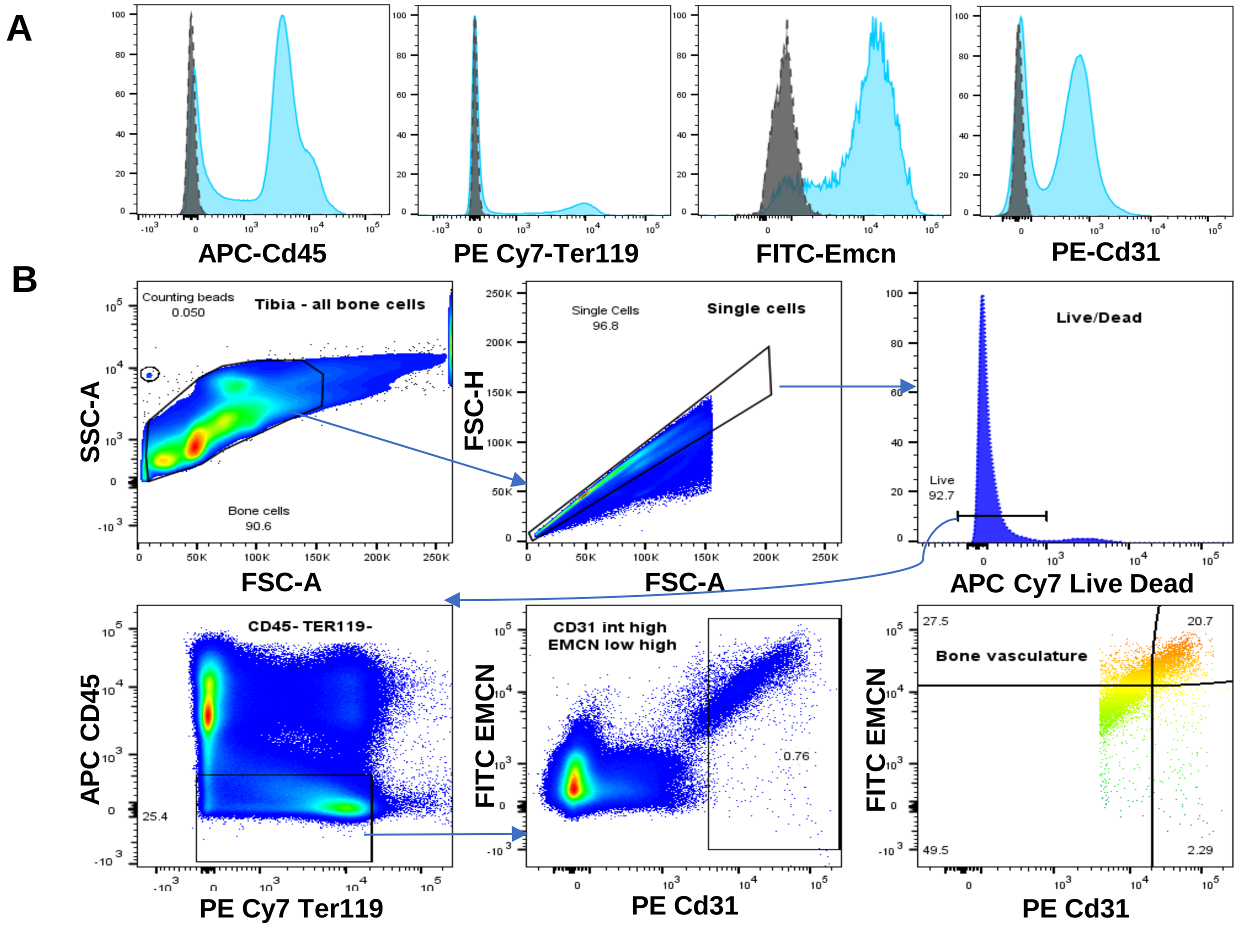

A) Histograms of antibody panel optimization, the grey traces represent staining with IgG isotype control and the blue traces represent staining with the indicated antibody, Cd45, Ter119, Emcn, Cd31 (left to right). **B)** Cells were gated based on size using SSC and FCS, before removing doublets based on FSC area vs FSC height. Live cells were gated based on the absence of viability dye staining, then Cd45 hematopoietic cells were gated out. Finally, Cd31^{high} Emcn^{high} ECs were defined based on expression of Cd31 and Emcn, positive cells were gated against fluorescence minus one controls.

Supplementary Table 1. PCR Primer list

Gene	Catalogue numbe	Company
Col1a1	Mm08801666	Taqman, UK
Ibsp	Mm00492555	Taqman, UK
Bglap	Mm03413826	Taqman, UK
